# Framework to Emulate Spacecraft Orbital Positioning Using GNSS Hardware in the Loop

**DOI:** 10.3390/s23020885

**Published:** 2023-01-12

**Authors:** David Forero, Segundo Esteban, Óscar Rodríguez-Polo

**Affiliations:** 1 Space Research Group, Polytechnic School, University of Alcalá, 28805 Alcalá de Henares, Spain; 2 Department of Computer Architecture and Automatic Control, Faculty of Physic Sciences, Complutense University of Madrid, 28040 Madrid, Spain

**Keywords:** satellite orbital simulation, satellite orbital determination, global navigation satellite system (GNSS), software defined radio (SDR), hardware in the loop (HIL)

## Abstract

The paper presents a framework to emulate spacecraft orbits using GNSS hardware in the loop that enables the evaluation of new orbital positioning algorithms. The framework software generates the spacecraft orbit and the GNSS signals, including the most common perturbations. These signals are modulated and transmitted by a software-defined radio and received by a commercial GPS receiver. The system is validated using a test orbit, where the GPS receiver accurately determines the spacecraft positions. Moreover, using raw data provided by the receiver, the spacecraft positions have also been determined by software for a low earth orbit, in which civil GPS receivers do not work.

## 1. Introduction

A Global Navigation Satellite System (GNSS) signal emulator is a valuable tool for the development of space systems. Its use facilitates the design and validation of the orbital control system of terrestrial satellites, as well as the Guidance, Navigation and Control (GNC) system of different types of spacecraft, including those aimed at lunar exploration [1,2]. Its scope of application can also be extended to other areas such as aviation, robotics, or ground, maritime and river navigation, offering a wide range of possibilities for both research and education.

In this context, it is particularly important to have test environments available to analyse the performance of a GNC system of any kind in the early stages of development. This is the main objective of this paper. Specifically, having a reliable emulator allows us to test, at a very low cost, different estimation algorithms, or the tolerance of a system against computer attacks, as well as many other scenarios involving GNSS.

To ensure the robustness of a GNC system, the environment must be sufficiently versatile and representative, allowing the analysis of the functionality, reliability and efficiency of the system, taking into account all the elements and sources of error that may affect the test [3], as well as the different scenarios that reproduce all the operational conditions.

In order to achieve results with a larger comprehension of real scenarios, it is essential to include as much GNSS system hardware and software as possible in the test. The final objective is to be able to validate the expected system behaviour, rigorously checking the result of the estimation and control algorithms that take the position provided by the GNSS receiver as the primary source of information. Including the GNSS receiver in this type of validation testing is key, as its performance and behaviour will propagate throughout the entire system.

According to this, the paper shows not only the framework design but its adaptation to the problem of satellite orbit estimation using a configurable Software Defined Radio (SDR) GNSS transmitter and commercial GNSS receivers. Specifically, the mitigation of perturbation introduced by the receivers hardware has been studied and a solution has been provided and evaluated by orbital estimation results. In this way, the framework has been used to identify and correct the error due to the receiver’s local clock bias.

In the article, an analysis of the state of the art on the use of GNSS in aerospace applications is first carried out. Then, the structure of the developed Framework (FW) is presented, both its mathematical foundation, as well as the hardware and software involved. The following sections explain in detail the two main FW subsystems and their hardware and software components. Next, the system is experimentally validated using an orbital position estimation problem, where the perturbation introduced by the receivers hardware is characterised and mitigated. Finally the conclusions are presented.

## 2. Related Works

In the last two decades, different Hardware-In-the-Loop (HIL) solutions have been proposed to evaluate performance of GNSS units, satellite formation navigation and estimation algorithms.

In 2009, Jae-Ik Park et al. established an HIL simulation test-bed to evaluate estimation and navigation algorithms [4]. In this development, however, the estimation is carried out using the carrier phase as the GNSS observable, and not the pseudorange, as proposed in our paper. Furthermore, their environment uses a commercial signal simulator, and does not offer the versatility of an SDR-based transmitter, which can be configured by file and thus work with any GNSS constellation, including those future constellations that will possibly be deployed in other environments, such as Mars or the Moon, for which open and configurable analysis environments will be required.

Wang Liduana et al. present, in 2010, a software-based GPS measurement simulator on L1 frequency and coarse acquisition (C/A) code for a space-oriented navigation system [5]. The simulator, coded in MATLAB language, generated both C/A code and carrier phase measurements. This work simulates and processes only the GPS signal and therefore does not generate the physical spread spectrum RF signals corresponding to the full GNSS constellation. Furthermore, it does not use real receivers and therefore does not take into account disturbances associated with the receiver hardware.

In 2015, Wang et al. showed that time and position data from mobile devices can be easily spoofed using very low-cost and open source tools [6]. This work shares with our paper the approach of using an SDR transmitter and low-cost hardware to build the environment. However, the objective pursued is different in that it does not address the complete problem of accurate position estimation of the receiving satellite, but rather the ability of the environment to fool commercial GNSS receivers under normal operating conditions over the Earth’s surface and at bounded velocities.

In 2016, Arul and Sudha designed a new and simple low-cost L1 GPS signal simulator to test and evaluate GPS receiver performance by software in a laboratory environment [7]. This simulation environment does not use HIL, so it does not facilitate the empirical evaluation of the effect of disturbances due to receiver electronics on the estimation of the orbital position and does not analyze the possible correction mechanisms to be applied.

In 2017, Peng developed a hardware testbed based on GNSS emulation for a group of spacecraft flying in low-altitude orbit with the ability to remotely detect the ionosphere [8]. This work addresses the same problem posed in this article: to have an environment that allows for emulating the reception of the GNSS signal and using it to determine the orbital position of satellites. However, as with the environment developed in [4], the use of a commercial GNSS signal simulator restricts its use, and does not allow the analysis of future GNSS constellations.

In 2018, Ebinuma developed a low-cost GPS simulator using software-defined radio, with which dry GPS attacks can be developed [9]. Finally, in 2020, Cao presented his PhD thesis on practical GPS spoofing attacks on consumer drones using the vulnerabilities of civilian GPS drones [10]. These two papers share the same environment approach based on the use of an SDR transmitter and commercial GPS receivers that has been used in our work, but as in [6], they do not address the problem of satellite orbital position estimation, and therefore do not offer a solution to the problem of using commercial receivers due to compliance with the Wassenaar agreement.

## 3. Framework Structure

### 3.1. Theoretical and Mathematical Basis of the Framework

The main objective of the FW is to generate the signals of a GNSS system to be received by a spacecraft (S/C) in space-time coordinates (t,x,y,z). GNSS positioning technology is based on the calculation by the receiver of the travel times of signals emitted from satellites in the GNSS constellation, whose position and time of emission are coded in the signal itself. To make this calculation possible, a common reference time, called GNSS time, has been defined, which has a constant offset to International Atomic Time (abbreviated TAI, from the French name Temps Atomique International), which in the equations we will denote by *t*. However, while the satellites of the GNSS constellations are equipped with redundant high-precision caesium or rubidium atomic clocks, the receiving spacecrafts use crystal oscillators that are not as accurate and sensitive to many disturbances, leading to various synchronisation problems. A modelling of these synchronisation problems can be found in [11,12]. Equation (1) shows the time of the GNSS constellation transmitters, which are synchronised with each other, as a function of their offset $\delta t^{s}\left(t\right)$. Equation (2) shows the receiver time as a function of its offset $\delta t_{r}\left(t\right)$.

(1) $$t^{s}\left(t\right)=t+\delta t^{s}\left(t\right)$$

(2) $$t_{r}\left(t\right)=t+\delta t_{r}\left(t\right)$$

The main observable is the direct measure of the travel time $t_{r}^{s}\left(t\right)$ of the P(Y) signal transmitted from the phase centres of the GNSS satellite antennas to the receiver. This time, as it is stated in [13], can be calculated by Equation (3) that contains the error introduced by clock offsets at both the transmitter and receiver. While P(Y) signal is encrypted, several techniques have been developed that allow observations to be made without the decryption hardware key [14]. In this case, there is a loss in the Signal/Noise ratio which results in a reduction in accuracy:

(3) $$t_{r}^{s}\left(t\right)=t_{r}\left(t\right)-t^{s}\left(t-\tau_{r}^{s}\left(t\right)\right)$$

where $t_{r}\left(t\right)$ is the local time at which the signal is received, transmitted from the GNSS satelite at its local time $t^{s}\left(t-\tau_{r}^{s}\left(t\right)\right)$, with $\tau_{r}^{s}\left(t\right)$ the true travel time. By replacing (1) and (2) in (3), Equation (4) is obtained.

(4) $$t_{r}^{s}\left(t\right)=\tau_{r}^{s}\left(t\right)+\delta t_{r}\left(t\right)-\delta t^{s}\left(t-\tau_{r}^{s}\left(t\right)\right)$$

Multiplying by the speed of light, we have in (5) the first approximation of the observable distance between the GNSS satellite and the spacecraft, called the pseudorange and denoted by $P_{r}^{s}\left(t\right)$, where the term $\rho_{r}^{s}$ in the equation represents the true distance:

(5) $$P_{r}^{s}\left(t\right)=\rho_{r}^{s}+c\cdot \left(\delta t_{r}\left(t\right)-\delta t^{s}\left(t-\tau_{r}^{s}\right)\right)$$

This equation does not yet take into account perturbations due to atmospheric effects, multi-path instrumental delays, or other effects such as thermal noise, although, in the case of low-orbit space missions, the only atmospheric effect that should be considered would be the ionospheric effect. This effect, defined in (6), is due to the delay of paths on the electromagnetic waves caused by ions and free electrons in the ionosphere, and depends on the frequency of the waves [12]:

(6) $$I_{r}^{s}\left(t,f\right)=\frac{40.3}{f^{2}}TEC_{r}^{s}\left(t\right)$$

where $TEC_{r}^{s}$ represents the total electron density along the signal path, and *f* is the frequency of waves.

The thermal noise $\epsilon_{rP}^{s}\left(t\right)$ is modelled in the literature as a random variable with zero mean. The rest of the perturbations, such as the instrumental delay $b_{rP}^{s}\left(t\right)$, the multipath $m_{rP}^{s}\left(t\right)$ and the system errors $S_{rP}^{s}\left(t\right)$, will be grouped in the term $M_{rP}^{s}\left(t\right)$ defined in Equation (7):

(7) $$M_{rP}^{s}\left(t\right)=b_{rP}^{s}\left(t\right)+m_{rP}^{s}\left(t\right)+S_{rP}^{s}\left(t\right)$$

Adding these perturbations, it is possible to compute the pseudorange required to build the GNSS signal, $\rho_{tx}\left(t\right)$, as shown by Equation (8):

(8) $$\rho_{tx}\left(t\right)=P_{r}^{s}\left(t\right)+I_{r}^{s}\left(t,f\right)+M_{rP}^{s}\left(t\right)+\epsilon_{rP}^{s}\left(t\right)$$

### 3.2. Framework Hardware and Software

**Figure 1:**
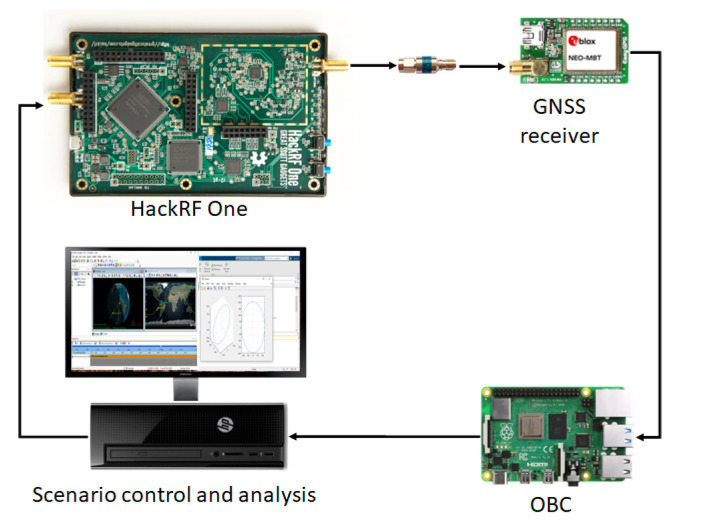
Hardware interconnection of GNSS emulation framework.

The FW physically consists of several hardware blocks in charge of emulating the spacecraft GNSS signals. The GNSS signal is generated by a low-cost Software Defined Radio (SDR) (HackRF One [15]) and received by a GNSS receiver (ublox NEO M8T [16]). The SDR is controlled by a personal computer. A software block, developed in Python and C languages, computes and transfers the baseband signal file for each S/C. The GNSS receiver is controlled by the On-Board Computer (OBC), in this case, a RaspBerry-Pi. Figure 1 shows a high-level view of this FW.

**Figure 2:**
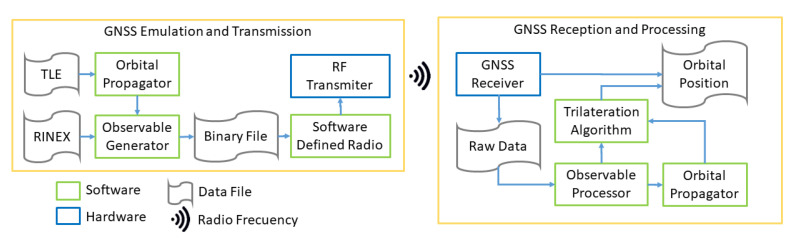
Functional diagram block of the framework.

The block diagram in Figure 2 shows more in detail the different functional blocks involved in the prototype.

As can be seen in the figure, one of the inputs to the functional model is a TLE (Two Line Elements) file. This file contains two lines of data encoding a list of orbital elements at a given epoch and is used by the FW to generate the spacecraft orbital trajectories using the SGP4 propagator [17].

The second input to the functional model is a navigation RINEX (Receiver Independent Exchange Format) file, which provides the GNSS constellation ephemeris data. The RINEX file is processed to generate the orbits of the constellation’s satellites at different epochs. Using the positions and velocities of the satellites relative to the spacecraft, it is possible to generate the observables to be captured by the GNSS receiver. These observables are encoded and modulated for radio frequency transmission using a SDR.

To finish the emulation chain, the radio signal is received and processed by the GNSS receiver in order to provide the orbital position. However, the receiver does not return any orbital position in a LEO orbit, due to the Wassenaar agreement. In this situation, it is necessary to work with raw data and process the observable by software.

## 4. GNSS Emulation and RF Transmission

As explained above, the GNSS emulator purpose is to generate, in real time, the RF signals that should be received by a spacecraft GNSS receiver. This block implements three software modules: the first two, named *Orbital Propagator* and *Observable Generator*, work together to generate the *Binary File* with the GNSS signal to be transmitted. The last module runs in a Software Defined Radio and configures the spread spectrum transmitter in charge of radiating this information. Each of these three software modules is explained in detail in the following subsections. One aspect to be taken into account is that orbital propagators normally work under the reference system known as Earth Center Inertial (ECI), while GNSS positioning algorithms normally use Earth Centered Earth Fixed (ECEF). For this reason, it will be necessary at several stages of the emulation to perform transformations between the two systems. In this FW, the transformations between the World Geodetic System (WGS 84), used by the GPS system, and the True Equator Mean Equinox (TEME) system, used by the SPG4 orbit propagator, have been implemented. In case of using other GNSS or propagators, appropriate transformations would have to be implemented.

### 4.1. Orbital Propagator

In order to emulate the GNSS signals that a spacecraft would receive during a mission, the first step is to calculate the spacecraft orbit. Since the FW has as its first reference scenario the GNSS emulation in near-Earth orbits, a Python implementation of the SGP4 propagator has been chosen to generate Low Earth Orbits (LEO). This propagator calculates the orbit of the spacecraft from the orbital parameters provided in a TLE file [18].

**Figure 3:**
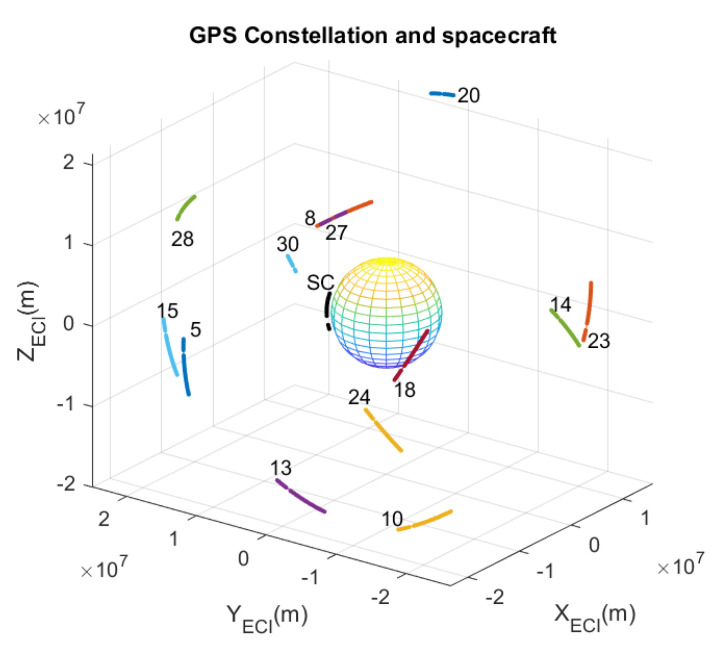
Orbital propagation of the S/C and GPS constellation.

For the propagation of the GNSS satellite constellation, a higher precision propagator is implemented in the Observable Generator module, which includes multiple corrections parameterised in the RINEX files. Figure 3 shows an example representation of the propagation of the orbits of the spacecraft and a GPS constellation using ECI as a reference system.

### 4.2. Observable Generator

As it was mentioned before, this module is in charge of generating the GNSS baseband to be transmitted. It uses as inputs the navigation RINEX file together with the spacecraft orbit file, previously calculated by the propagator. The output provided by this module is a binary file with the baseband that must be transmitted by the radio and received by the spacecraft GNSS receiver.

In order to provide a realistic emulation, the Observable Generator integrates the ability to incorporate perturbations that can be enabled and disabled via software. As previously described, GNSS receivers suffer from perturbations due to tropospheric, ionospheric and relativistic effects, as well as inaccuracies in their internal clocks and instrumental delays. Most of the perturbations are modelled in the RINEX file, including: the clock synchronisation parameters (toc) $a_{0}$, $a_{1}$ y $a_{2}$, the instrumental delay manipulation tgd and the ionospheric parameters $\left(\alpha_{1},...,\alpha_{4}\right)$ and $\left(\beta_{1},...,\beta_{4}\right)$.

The pseudo-code Listing 1 shows the implementation of the GNSS Observable Generator. In the initial stages, the ReadRINEXFile and ReadOrbitFile functions read the inputs, information of GNSS constellation and the orbit of the spacecraft to be emulated. This is followed by the configuration of the active perturbations to be processed, the buffer allocation and the initialization of the channels. Then, a loop is entered which simulates the relative positions of the transmitting satellites and the spacecraft. In this loop, for each SC position of the orbit the GNSS positions are retrieved using GetGNSSEphemeris function, the visible satellites are determined in DetermineVisibleGNSSs function, and the observable signals are generated by ObservableGenerator function. AddPerturbations function adds modification to the observable signals for each active perturbation. Finally, GenerateBasebandSignal function generates the final signal, and it is added to the output binary file. This generator is implemented in C, and a Python wrapper has been built to integrate it into the simulator.

**Listing 1.** GNSS signal generator.
Initialization ()

ReadRINEXFile ()

ActivePerturbation ()

InitializeBuffer ()

InitializeChannels ()

While (not end S/C orbit)

{

  GetGNSSEphemeris ()
  DetermineVisibleGNSSs ()  ObservableGenerator ()  AddPerturbations ()  GenerateBasebandSignal ()  AppendToBinaryFile ()
}


**Figure 4:**
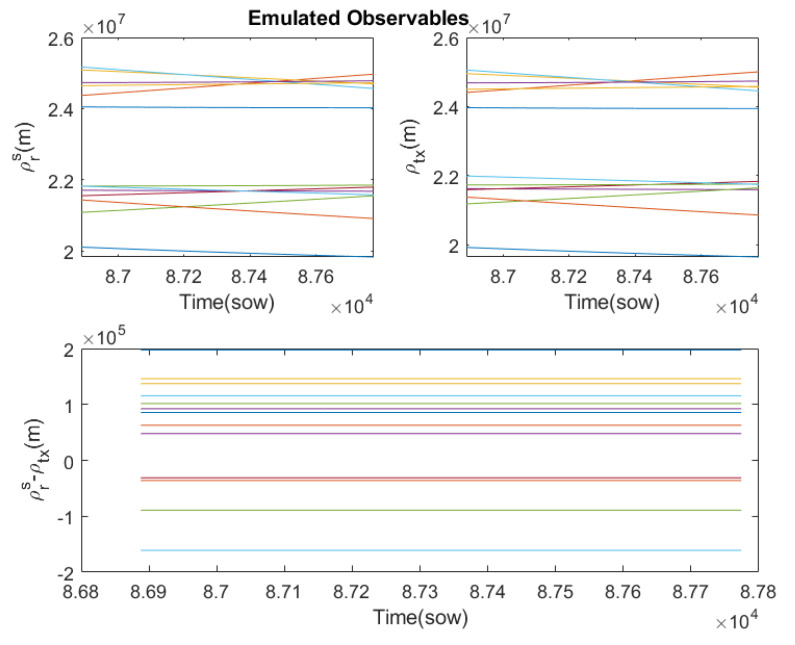
Difference between true path and pseudoranges used in the transmission for the GPS constellation (each color represents a different GPS emitter).

Figure 4 compares the true path, $\rho_{r}^{s}\left(t\right)$, in the left graphic, and the pseudoranges used in the transmission, $\rho_{tx}\left(t\right)$, in the right graphic. Although, due to the scale, they look quite similar, and their differences can be seen in the graph below. According to Equation (8), these differences are mainly due to the travel time, the clock error of GPS transmitters and the instrumental delay of the GPS transmitters, since atmospheric disturbances are negligible, and thermal noise has been disabled. This graph already shows that each pseudorange is perturbed in a different way. For the case of space orbits, the ionospheric and tropospheric effects can be discarded, but the rest of the effects cannot. In particular, instrumental delay, clock biases and the relativistic effect, due to the high orbital velocities, are quite relevant. The transmitted pseudoranges are very important in this simulator because they indirectly define the moment at which the radio signals should be transmitted to the S/C because the receiver is wired to the radio.

### 4.3. Software Defined Radio

**Figure 5:**
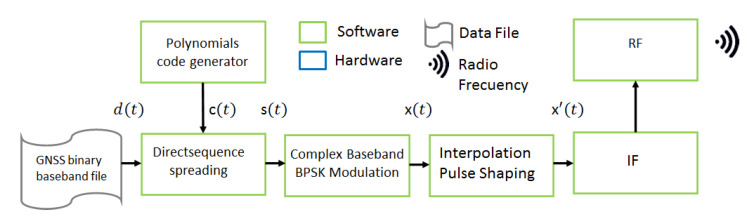
Functional block diagram of a simple DSSS modulator.

Software defined radio (SDR) is a radio communication system where the typical hardware components of a radio system are implemented in software. In this project, GNU Radio software toolkit is used [19]. The software produces a signal identical to that transmitted by the GNSS constellation. This signal is generated in the digital domain and then converted into an analogue signal in the time domain. Finally, it is radiated by means of an antenna or a guided medium to the GNSS receiver. The SDR implemented in this project is a simple Direct Sequence Spread-Spectrum (DSSS) modulator represented in the block diagram in Figure 5.

The elements involved in the DSSS modulator are:

- GNSS binary baseband file, with output d(t), is the binary file with the observable encoding generated by the *Observable Generator* routine.
- Polynomials code generator, with output c(t), implements a shift register with linear LFSR feedback to generate pairs of predefined sequences representing a six degree polynomial as a random source of the polynomial generator.
- Direct sequence spreading, with output s(t), performs a spread spectrum modulation of the signals d(t) and c(t).
- Complex Baseband BPSK Modulation, with output x(t), is a binary phase shift keying (BPSK) modulator at spread spectrum.
- Interpolation Pulse Shaping, with output $x^{{}^{\prime }}\left(t\right)$, interpolates each BPSK symbol with K samples according to a rectangular pulse to transform the baseband to an intermediate frequency.
- IF Stage converts the interpolated signal to an intermediate frequency analog signal.
- RF Transmit adds the carrier to convert the system into a radio transceiver.

### 4.4. Hardware Tx

The radio used in the FW is a HackRF One. It is a low-cost SDR peripheral with the capacity of transmitting or receiving radio signals, from 1 MHz to 6 GHz, designed to enable the testing and development of radio technologies [19]. The main characteristics of HackRF One are:

- Operating frequency 1 MHz a 6 GHz;
- Half-duplex transceiver;
- Twenty million samples per second;
- Eight bit of quadrature samples (I y Q);
- Receive and transmit gain configurable for software;
- The antenna connector is an SMA.

**Figure 6:**
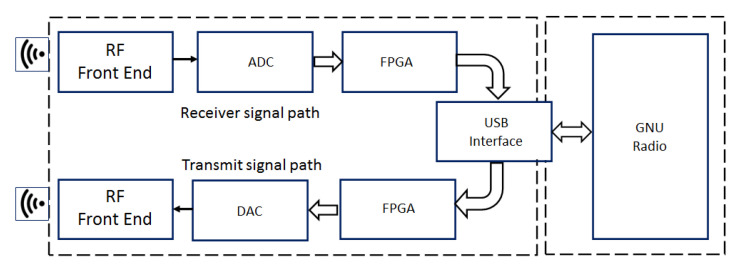
HackRf One main components: Radio Frequency interface (RF Front End), Analog to Digital Converter (ADC) and Digital to Analog Converter (DAC), Field-Programmable Gate Array (FPGA) and a USB interface connected to the GNU Radio.

The hardware of the radio is very simple, and Figure 6 shows a block diagram with the main components. The host computer runs the SDR code and produces digital signal samples at a sampling rate lower than the ADC/DAC rate. The FPGA uses the data sample stream from the host computer and performs high sample rate signal processing to make the resulting digital signal compatible with the ADC/DAC requirements. The high sample rate processing is performed in the FPGA, while the low sample rate processing is performed in the host computer running the SDR algorithms.

## 5. GNSS Reception and Processing

At this stage, it is possible to work with commercial or ad hoc designed GNSS receivers. The *GNSS Receiver* hardware can either determine the position directly or provide the *Raw Data* to be processed by software blocks: *Observable Processor*, *Orbital Propagator* and *Trilateration Algorithm*.

### 5.1. GNSS Receiver

To validate the simulator, the commercial GNSS receiver NEO-M8T from Ublox [16] has been used. It is a medium performance receiver, compatible with the BeiDou, GLONASS, Galileo and GPS constellations. This receiver is limited by the Wassenaar agreement, which restricts the operation of GNSS receivers to altitudes below 50,000 m and velocities up to 500 m/s. Because of this, it does not determine the position for space orbits. In this case, the receiver NEO-M8T provides the raw measurements shown in Table 1.

### 5.2. Processing

To process the raw data, a software GNSS receiver with three stages has been implemented:

- *Observable Processor*: To estimate the true distances, $\rho_{t}^{s}\left(t\right)$, the corrections of the different perturbations are added to the pseudoranges measured by the receiver, $\rho_{rx}\left(t\right)$. These estimates are called processed pseudoranges, $\rho_{proc}\left(t\right)$, which are shown in Table 1. With these values, the travel times and the transmission times, $t{}^{s}\left(t\right)$, can now be estimated.
- *GNSS Orbital Propagator*: The GNSS satellites orbits are propagated using transmission times and ephemerides of the constellation. In Figure 3, the orbits of GPS satellites have some discontinuities when the receiver has not registered a signal for those GPS satellites.
- *Trilateration Algorithm*: The GNSS satellite positions and the processed pseudoranges are the inputs of a trilateration algorithm to calculate the position of the spacecraft. As a use case, an algebraic solution of trilateration problem has been implemented [20], but many others algorithms may be evaluated.

## 6. Framework Validation

Next, a step-by-step validation of the developed environment is carried out. For this purpose, the GPS constellation has been emulated and different orbits for the S/C have been evaluated using the developed FW.

### 6.1. Validation of GNSS Emulation and Transmission

The first stage is to validate the GNSS Emulation and Transmission stage. For this purpose, the GPS receiver has been used as a black-box to check whether it understands the signals emitted by the emulator and calculates the orbital positions correctly. In order for the receiver to determine the position, it is necessary to work with an orbit that complies with the Wassenaar agreement. Therefore, it has been necessary to introduce a small modification in the orbital propagator that allows us to modify two orbital parameters, the semi-major axis and the mean momentum of the orbit. Thanks to the fact that SPG4 is a Kepler propagator, the orbit, although lower and slower, follows geometrical laws and does not fall on the Earth’s surface.

**Figure 7:**
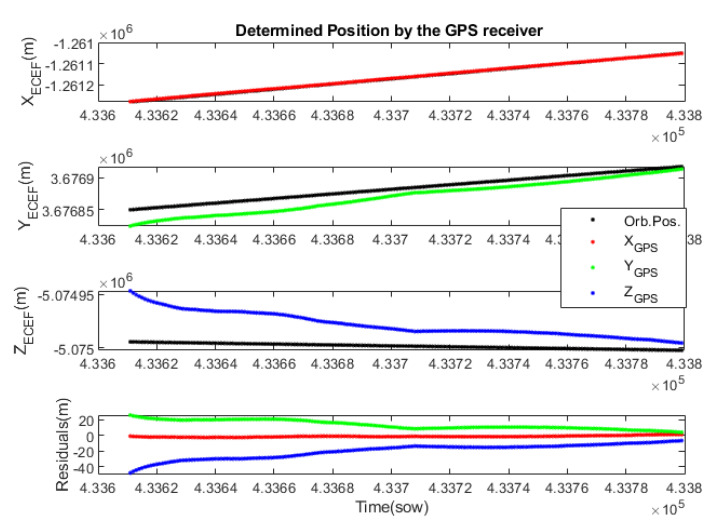
Position determined by the GPS receiver for the modified orbit. The ECEF coordinates of the propagated orbit are shown in black and those determined by the GPS in red, green and blue. The last graph shows the evolution of the residuals or errors.

In this case, the FW generates the radio signals from the modified orbit of the S/C, and the GPS receiver is fed with these signals. After a few seconds of initialisation, the GPS receiver determines the S/C’s position and orbital velocity. Although this receiver has Spoofing detection, it does not detect anything anomalous. Figure 7 shows the orbital positions propagated by the FW and the orbital position determined by the GPS receiver and their residuals. The GPS is using a Kalman filter to smooth the noise, but also introduces a transitory behaviour. After the transitory, the residuals are within the technical specifications of the GPS receiver.

These results validate the GNSS emulation and transmission stage, as the signals are understood by a commercial receiver. Furthermore, it appears that the accuracy of the radio used is more than sufficient to generate synthetic GNSS signals.

**Figure 8:**
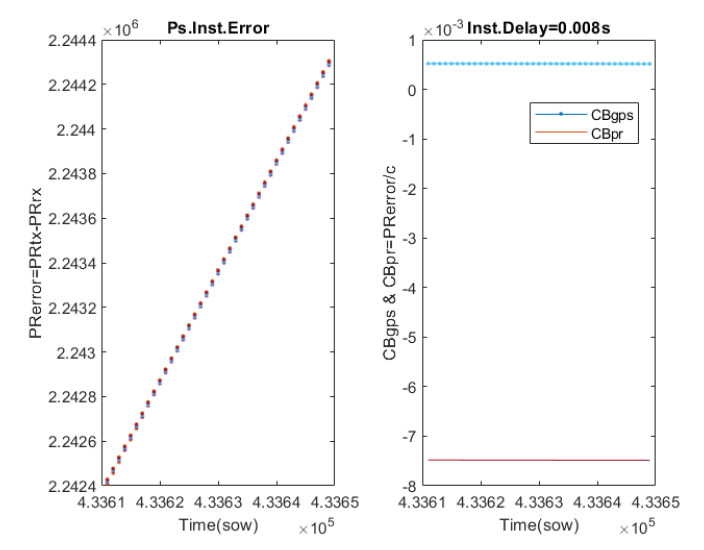
Estimation of the instrumental delay of the GPS receiver. The left plot shows the error between the pseudoranges used by the radio to transmit and those recorded by the GPS (each color represents a different GPS emitter). The right plot shows the Clock Bias estimated by the GPS and those calculated from the error between the pseudoranges.

Figure 8 left shows the difference between the pseudoranges used to transmit, $\rho_{tx}$, and those recorded by the GPS, $\rho_{rx}$. Theoretically, these should be coincident, but instrumental errors cause them to differ. It can be seen that the error evolves over time, but coincides for all GPS transmitters, as the lines overlap. This indicates that this error is mainly due to the GPS receiver, and specifically, it has been identified as an instrumental error associated with the Local Clock Bias (CB) of the receiver, which suffers from a drift. The GPS receiver estimates the CB, through its Trilateration algorithm. Figure 8 right shows the CB estimated by the GPS receiver, CBgps, and the pseudorange errors expressed in seconds, CBpr. It can be seen that there is a difference of 8 ms, which can be interpreted as an instrumental time delay of the GPS receiver.

This FW allows for characterising the instrumental delay of a GPS receiver, which is very difficult to do in flight because the GPS excitation signal is not available. In this process, the synchronisation of the signals from the transmitting stage and the signals from the receiving stage is critical. To solve this problem, the simulator time stamps each signal. This delay is critical for space applications, as the orbital velocities are very high. In the case studied, an instrumental delay of 8 ms has been detected, which would be an error of the order of 50 m under the conditions of a LEO orbit, with an orbital velocity of about 21,000 km/h.

### 6.2. Validation of GNSS Reception and Processing

To check that the FW allows the evaluation of other determination or trilateration algorithms, it is necessary to work at a lower level, using the raw signals recorded by the GPS receiver. The results presented below correspond to the LEO orbit shown above in Figure 3. For this orbit, the GPS receiver we are using does not determine the position, but provides raw signals from the observables, which are stored by the FW in the *RawData* file.

**Figure 9:**
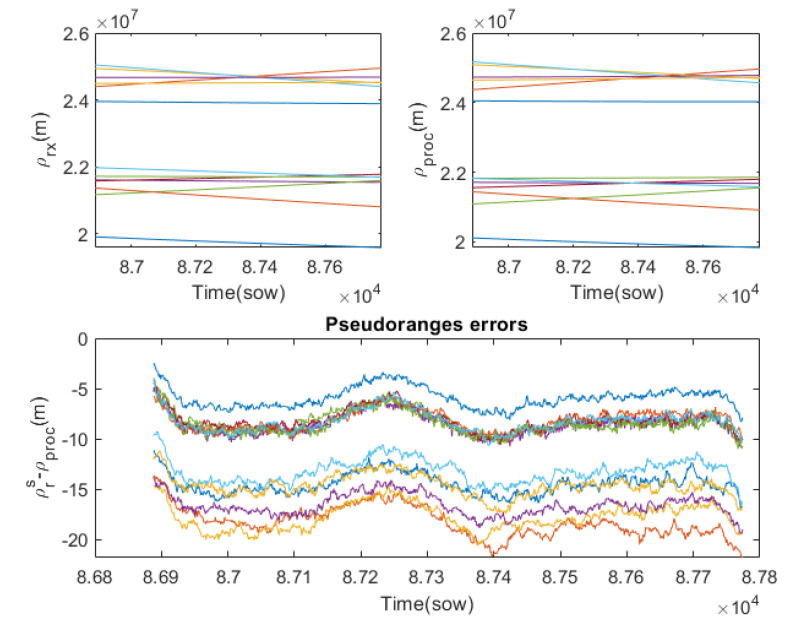
Received pseudoranges and processed pseudoranges and their errors (each color represents a different GPS emitter). **Top left** plot shows the pseudoranges measured by the receiver. **Top right** plot shows the processed pseudoranges. **Bottom** plot shows the difference between true distances and the processed pseudoranges.

From the raw signals of the observables, the first step is to process the received pseudoranges to estimate the true distances, called the processed pseudoranges. This can be achieved using the formalism described in Section 3.1. In this observable processing stage, it is also possible to cancel those disturbances that are parameterised in the RINEX files. Figure 9 shows in the first two plots the raw pseudoranges, $\rho_{rx}$, and the processed ones, $\rho_{proc}$. Due to the scale they look similar, but they actually show significant differences. Figure 9 bottom shows the difference between the true distances and the processed pseudoranges. Although the thermal noise disturbance has not been included, a negligible noise can be observed which is due to the transmission-reception channel used. Thanks to all the corrections made, an acceptable error in the processed pseudoranges of less than 20 m is achieved. Ideally, these errors should be practically zero, but instruments are not perfect and make measurement errors. Because the signals from the GPS transmitters arrive at different times at the receiver, the temporal resolution of the receiver affects them differently. Due to the high orbital velocities, small temporal errors translate into errors of the order of metres. The developed FW has again highlighted the limitations of the GPS receiver used.

The emission times are calculated from the processed pseudoranges and the reception times. The orbital propagator then calculates the positions of the GPS transmitters. Then, a trilateration algorithm is applied to determine the orbital position of the S/C and its local clock bias. In this case, an extended version of the [20] algorithm has been implemented, which also estimates the clock bias.

**Figure 10:**
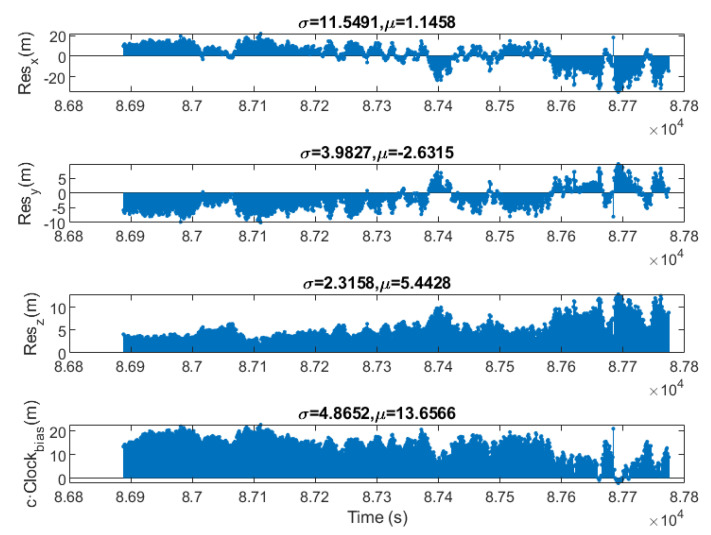
Residuals of the determined position by software for a LEO Orbit.

Figure 10 shows the non filtered residuals of the S/C determined coordinates with respect to the propagated ones. The bottom graph shows the distance error associated with the determined local clock bias. Since the instrumental delay of the GPS receiver has been eliminated, the distance error associated with the clock bias is quite low. The noise is mainly due to receiver hardware limitations and transmission channel noise.

## 7. Conclusions

This paper presents a low-cost framework for hardware-in-the-loop orbit simulation. The framework allows validation of the integrated behaviour of the hardware and software involved in orbit position determination. It includes the simulation of perturbations due to ionospheric effects, tropospheric effects, thermal noise and instrumental delay of emitter and receiver, providing a realistic recreation of aerospace and hardware environments. The framework uses as input information the spacecraft’s orbital parameters, provided through a TLE file, and the GNSS constellation ephemeris data, provided through the RINEX file. From this information, the system calculates the GNSS constellation signals to be received by the spacecraft and, using a software-defined radio, transmits them to a GNSS receiver. The position determined by the GNSS receiver can be evaluated directly, thus employing the receiver as a black-box that directly calculates the position of the spacecraft during the simulation. In addition, a second way of using the framework gives access to the raw observables from the GNSS receiver and uses them to evaluate a particular determination algorithm.

Initial validation of all stages of the framework has been completed using black-box mode on the NEO-M8 commercial GNSS receiver connected in baseband to the software defined radio. The results obtained show that the framework works as expected with position determination accuracy in the order of the technical specifications of the receiver. Subsequently, tests have been completed using, this time, the raw observables provided by the receiver, thus validating the orbital position determination algorithm. The results show that the developed framework, although low-cost, allows the evaluation of orbital positioning systems at different levels. The environment is useful for verifying the performance of commercial GNSS receivers and checking the quality of their estimates. In this sense, it has been possible to verify that the NEO-M8 GNSS receiver, complying with the Wassenaar agreement, does not directly offer position determination outside the permitted limits, although it does allow access to the raw observables.

The framework provides a very useful tool for evaluating small satellite missions that require precise orbital position determination, such as those formed by satellite constellations, where in-flight formation and collision warning systems need to be managed. At orbital velocities, instrumentation errors significantly influence the position determined. Furthermore, how measurement errors are propagated to the determined position depends very much on the algorithm used and the configuration of the selected GPS satellites. The framework presented in this paper is ideal for qualifying the robustness of new algorithms against instrumental errors and external disturbances.

Furthermore, the modularity and adaptability of the developed environment make it easy to extend its use to other scenarios, such as redundant positioning systems that combine beacons fixed on the Earth’s surface with information from the GNSS satellite constellations themselves. They also enable the simulation of the deployment of future navigation systems for the Moon or Mars, or even to provide coverage for deep space exploration missions.

Future work includes extending the capabilities of the framework to work in real time, and developing a GNSS receiver using a software-defined radio. This receiver will overcome the restrictions of commercial receivers, both in orbit height and spacecraft speed, and thus provide the positioning of the spacecraft in real environments, without the need to process raw observables. In addition, to complete the capabilities of the framework, it is planned to include simulation parameters related to space weather, such as radiation and pressure perturbations, and to develop other orbit determination algorithms.

## Figures and Tables

**Table 1:** Raw data of GPS receiver: reception time in Second Of Week (SOW), transmitter identifier (ID), the pseudorange measurement ($\rho_{rx}$), the phase carrier measurement ($\phi_{r}^{s}$) and the Doppler measurement or zero crossings of the received frequency. The column $\rho_{proc}\left(m\right)$ is the processed pseudorange.

SOW $t_{r}\left(s\right)$	ID	$\rho_{\mathrm{rx}}\left(m\right)$	$\phi_{r}^{s}\left(\mathrm{Hz}\right)$	Doppler	$\rho_{\mathrm{proc}}\left(m\right)$
432,381	G10	23,114,092.3772	107,533,978.113	−1009.187	23,114,092.3772
432,381	G24	22,062,373.2656	102,007,202.204	1776.915	22,160,204.6500
43,2381	G13	20,289,894.7344	926,927,48.9384	1532.4572	20,438,859.9648
432,381	G15	23,910,225.0839	111,717,657.7537	−1215.151	23,999,154.3198

## Data Availability

Not applicable.

## Abbreviations

The following abbreviations are used in this manuscript:

ADC	Analog to Digital Converter
BDS	BeiDou Navigation Satellite System
DAC	Digital to Analog Converter
DSSS	Direct-Sequence Spread Spectrum
ECEF	Earth-Centered, Earth-Fixed
ECI	Earth-Centered Inertial
FPGA	Field-Programmable Gate Array
FW	Framework
GNSS	Global Navigation Satellite System
GNC	Guidance, Navigation and Control
HIL	Hardware In the Loop
LEO	Low Earth Orbit
RF	Radio Frequency
RINEX	Receiver INdependent EXchange
S/C	Spacecraft abbreviated
SDR	Signal/Software Defined Radio
SPG4	Simplified General Perturbations Satellite Orbit Model 4
TEC	Total Electron Content
TLE	Two Line Element
TDI	Travelling Ionospheric Disturbances
